# A Software for Calculating the Economic Aspects of Floating Offshore Renewable Energies

**DOI:** 10.3390/ijerph17010218

**Published:** 2019-12-27

**Authors:** Laura Castro-Santos, Almudena Filgueira-Vizoso

**Affiliations:** 1 Departamento de Enxeñaría Naval e Industrial, Escola Politécnica Superior, Universidade da Coruña, Esteiro, 15471 Ferrol, Spain; 2 Departamento de Química, Escola Politécnica Superior, Universidade da Coruña, Esteiro, 15471 Ferrol, Spain; almudena.filgueira.vizoso@udc.es

**Keywords:** feasibility study, offshore wind, levelized cost of energy (LCOE), wave energy, software

## Abstract

The aim of this work is to develop a software to calculate the economic parameters so as to determine the feasibility of a floating offshore renewable farm in a selected location. The software can calculate the economic parameters of several types of offshore renewable energies, as follows: one renewable energy (floating offshore wind—WindFloat, tension leg platform (TLP), and spar; floating wave energy—Pelamis and AquaBuoy), hybrid offshore wind and wave systems (Wave Dragon and W2Power), and combined offshore wind and waves with different systems (independent arrays, peripherally distributed arrays, uniformly distributed arrays, and non-uniformly distributed arrays). The user can select several inputs, such as the location, configuration of the farm, type of floating offshore platform, type of power of the farm, life-cycle of the farm, electric tariff, capital cost, corporate tax, steel cost, percentage of financing, or interest and capacity of the shipyard. The case study is focused on the Galicia region (NW of Spain). The results indicate the economic feasibility of a farm of floating offshore renewable energy in a particular location in terms of its costs, levelized cost of energy (LCOE), internal rate of return (IRR), net present value (NPV), and discounted pay-back period. The tool allows for establishing conclusions about the dependence of the offshore wind resource parameters, the main distances (farm–shore, farm–shipyard, and farm–port), the parameters of the waves, and the bathymetry of the area selected.

## 1. Introduction

Floating offshore renewable energies are those that are installed in deep waters (more than 50 m). They are composed by several main components, namely: energy generators, floating offshore platforms, mooring, anchoring, and electric systems. The main difference between these floating platforms and the fixed platforms (up to 50 m of depth; monopiles [1], tripiles, jackets [2], etc.) [3] is that these last ones have platforms fixed to the seabed using several devices; therefore, they do not have mooring and anchoring systems. 

In this context, the most developed technology is offshore wind systems, although wave energy will have great development in the future too. Floating offshore wind energy has been developed over the last years, mainly in Europe and Japan [4,5]. Hywind Scotland was the first commercial floating offshore wind farm installed in the world. It was installed in 2017 in Scottish waters [6] by Statoil. It is based on a spar floating platform called Hywind, which was previously probed in Norway in 2009 [7]. It has five spar platforms that were built in the Navantia-Fene shipyard in Fene (A Coruña, Spain). This shipyard is the leader in floating offshore wind building—it built one platform for the WindFloat Atlantic project (Windplus) in Portugal in 2019 (the first floating offshore wind farm in the Iberia Peninsula [8]), and it is starting to build five platforms for the Kinkardine project (Aberdeen, U.K.), consisting of 9.5 MW platforms [9].

Floating offshore renewable energies will have a great future. However, nowadays, they are still in development, mainly wave energy systems and hybrid technology, and need to increase their unitary power in order to be more competitive with conventional offshore wind. Therefore, it is important to determine the best areas where a floating offshore renewable energy farm can be installed, and calculate their economic feasibility in order to make decisions about the final installation of these technologies. There are some studies about the types of restrictions of the areas where offshore wind energy is installed [10], restrictions for wave energy [11], mapping the electric system of offshore ports [12], and a comparison between onshore and offshore wind [7]. 

There is a lot of information about offshore platforms, namely: semisubmersible [13,14,15], tensioned leg platform (TLP) [16,17], and spar [18]. Arapogianni [19] analysed the main floating systems and differences between the grid connected systems (Hywind of Statoil and WindFloat of Principle Power) and the concepts under development (Advanced Floating Turbine of Nautica WindPower; Aero-generator X of Wind Power Ltd. Arup, Azimut of Consortium of Spanish Wind Energy Industry lead by Gamesa; Blue H TLP of Blue H; DeepCWind floating wind of the consortium of the University of Maine, AEWC, Seawall, Maine Maritime Academy, Technip, NREL and MARIN; Deepwind, which is an EU project; DIWET Semisub of Pole Mer; EOLIA of Acciona Energy; IDEOL of IDEOL; GICON TLP of GICON; Hexicon platform of Hexicon; HiPRwind, an EU project; Karmoy of Sway; Ocean Breeze of Xanthus Energy; W2Power of Pelagic Power; Pelastar of Glosten Associates; Poseidon Floating Power of the Floating Power; Sea Twirl of Sea Twirl; Trifloater Semisub of Gusto; Vertiwind of Technip and Nenuphar; WindSea Floater of Force technology NLI; Winflo of Nass and Wind and DCNS; ZEFIR Test Station of Catalonia Institute for Energy Research; and Haliade of Alstom). Perhaps the most representative is the research developed by Jonkman and Matha [19,20], where a spar, a semisubmersible, and a TLP (tensioned leg platform) were calculated, because it compares the three main types of floating offshore wind platforms. In this context, Sclavounos et al. [21,22] also considered the taught leg buoy concept. Collu et al. compared fixed and floating structures for a 5-MW wind turbine [23].

However, all of these studies did not take into account the economic aspects of such technologies, although Wind Europe has established the importance of cost reductions [24] in order to create competitive technologies comparable with onshore renewable energies. In addition, presently, there is not any software that allows the user to develop the economic calculation of the offshore renewable energy of several choices (wind, waves, and wind and waves), which is the objective of the present work.

The aim of this paper is to create a software to calculate the most important parameters of the economic feasibility of a floating offshore renewable farm in a selected location. 

## 2. Software Characteristics

### 2.1. Description of the Software

The software can calculate the economic parameters of several types of offshore renewable energies, as follows: one renewable energy (floating offshore wind—WindFloat, TLP, and spar; floating wave energy—Pelamis [25] and AquaBuoy [26]), hybrid offshore wind and wave systems (Wave Dragon [27] and W2Power [28]), and combined offshore wind and waves with different systems (independent arrays, peripherally distributed arrays, uniformly distributed arrays, and non-uniformly distributed arrays). The user can select several inputs, namely: location, configuration of the farm, type of floating offshore platform, type of calculation of the wave’s energy, power of the farm, life-cycle of the farm, electric tariff, capital cost, corporate tax, steel cost, percentage of financing, interest, and capacity of the shipyard. The case study is focused on the Galicia region, located in the North-West of Spain. The economic results are as follows: the cost of each phase of the life-cycle of the project, the total cost of the life-cycle of the farm, the internal rate of return (IRR), the net present value (NPV), the discounted payback period (DPBP), and the levelized cost of energy (LCOE). The results indicate the economic feasibility of a farm of floating offshore renewable energy in a particular location.

The objective of the created software (W2EC by LCS “Wind and Wave Energy farm economic Calculator” by Laura Castro Santos) is to calculate several economic parameters of a floating offshore renewable energy farm in a location. The formulation of costs has been previously developed [28]. This software has been created for the locations of Galicia, the Galicia and Cantabric region, and Portugal, because these are the input maps data that we have available. However, if the data of other locations is obtained, new areas of analysis can be included. Therefore, the software can be used for any location that the user wants. The software has been registered.

The user can select the inputs wanted in order to calculate the economic maps of the location selected. The programming language is MATLAB® (MathWorks, Natick, MA, USA), and the software is compatible with Microsoft Windows® (Microsoft, Redmond, WA, USA).

Firstly, the software calculates the main costs of the life-cycle of the offshore renewable energy farm (definition of the concept, design and development, manufacturing, installation [13], exploitation, and dismantling), and then the total life-cycle cost of the farm is calculated [29,30]. The novelty of this work is to develop a software using an easy interface and considering several types of offshore renewable energies. Therefore, the calculation of the costs will be conditioned by the type of energy selected, among other factors.

In addition, the map inputs (scale parameter of the offshore wind resource, shape parameter of the offshore wind resource, distance from farm to shore, distance from farm to shipyard, distance from farm to port, height of waves, period of waves, and bathymetry) generate a map of the energy produced in the specific location selected.

### 2.2. Inputs of the Software

**Figure 1:**
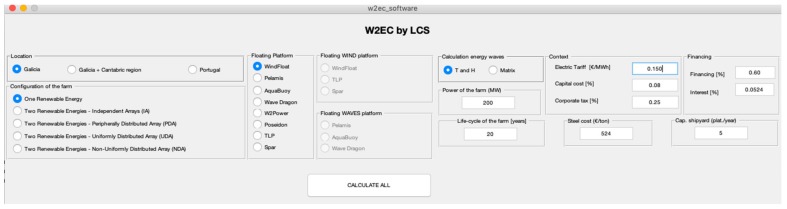
Software interface.

**Figure 2:**
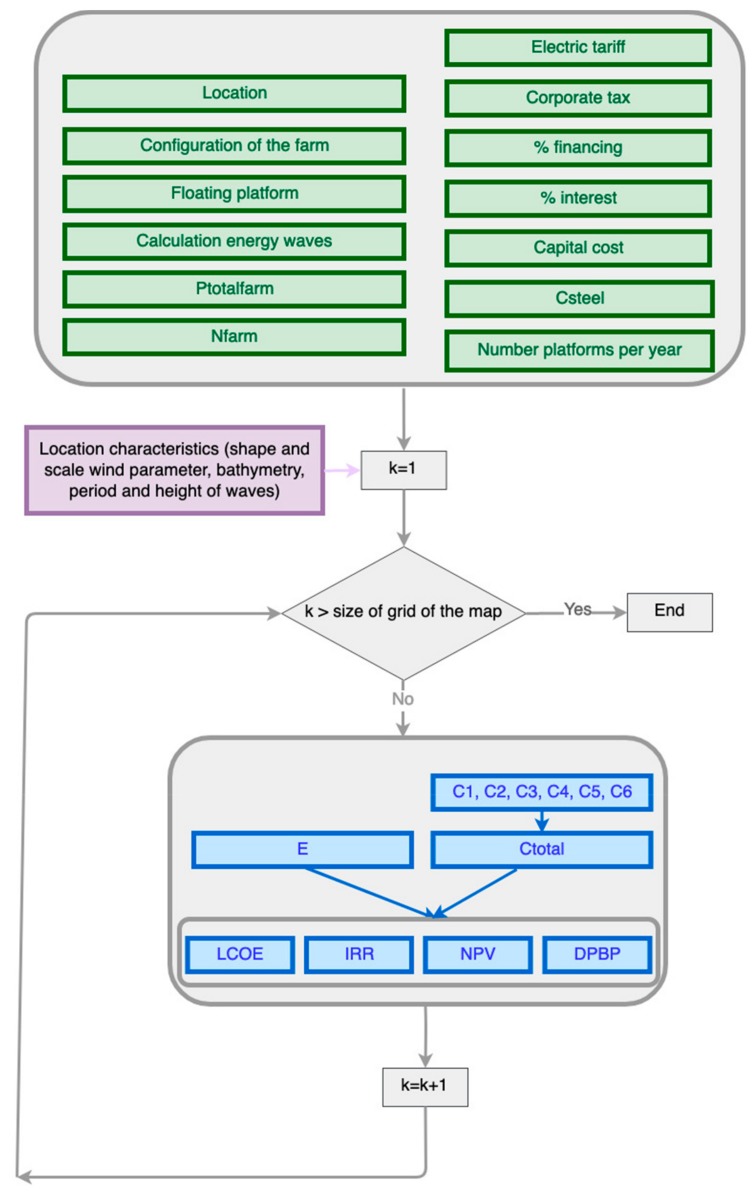
Flow diagram of the software. Input variables being the following: the total power of the farm (Ptotalfarm), number of years of the life-cycle (Nfarm), cost of steel (Csteel), the number of points that the input maps have (k), energy produced (E), total cost of the farm (Ctotal), defining cost (C1), designing and developing cost (C2), manufacturing cost (C3), installing cost (C4), exploiting cost (C5), and dismantling cost (C6).

In this context, the inputs of the software are (see Table 1 and Table 2 and Figure 1):

Figure 2 shows the input variables (location, configuration of the farm, floating platform, calculation of energy waves, total power of the farm (Ptotalfarm), number of years of the life-cycle (Nfarm), electric tariff, corporate tax, % financing, % interest, capital cost, cost of steel (Csteel), and number platforms per year) that are used to calculate the total cost of the farm (Ctotal) and the input maps related to the selected location (shape and scale of the wind parameter, bathymetry, and period and height of waves), whose value is different depending on the point (k) of the defined grid.

The software calculates the economic parameters of the project, considering the energy produced and the total life-cycle cost such as input variables. The economic results obtained are as follows:

Internal rate of return of the financed project (IRR FP; %).
Net present value of the financed project (NPV FP; M€).
Discounted payback period of the financed project (DPBP FP; years).
Levelized cost of energy (LCOE; €/MWh).

### 2.3. Criteria and Protocol

**Figure 3:**
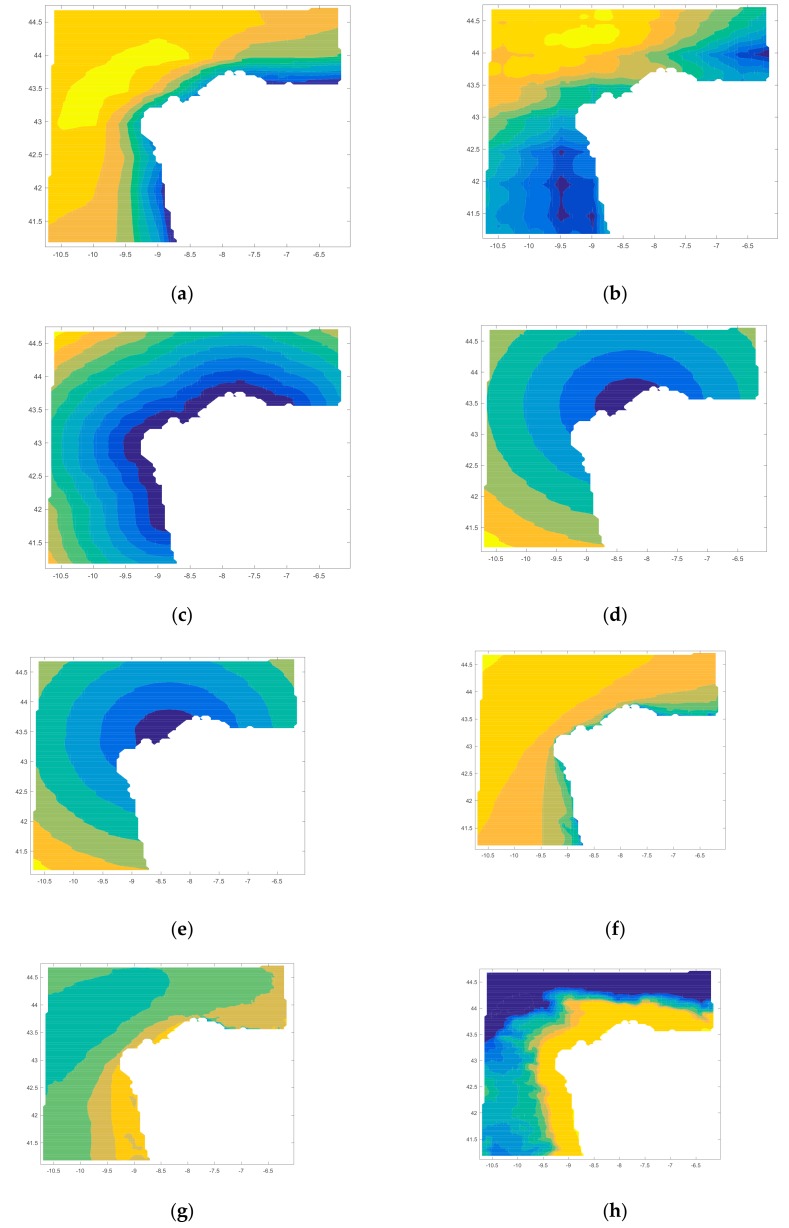
Map inputs: scale parameter of the offshore wind resource (**a**) in m/s, shape parameter of the offshore wind resource (**b**), distance from farm to shore in m (**c**), distance from farm to shipyard in m (**d**), distance from farm to port in m (**e**), height of waves in m (**f**), period of waves in s (**g**), and bathymetry in m (**h**).

Regarding the location, the user selects this input and he has three options (Galicia, Galicia + Cantabric region and Portugal). These choices condition the map files (in .mat) that the software selects for acting related to scale parameter of the offshore wind resource (Figure 3a), shape parameter of the offshore wind resource (Figure 3b), distance from farm to shore (Figure 3c), distance from farm to shipyard (Figure 3d), distance from farm to port (Figure 3e), height of waves (Figure 3f), period of waves (Figure 3g) and bathymetry (Figure 3h).

**Figure 4:**
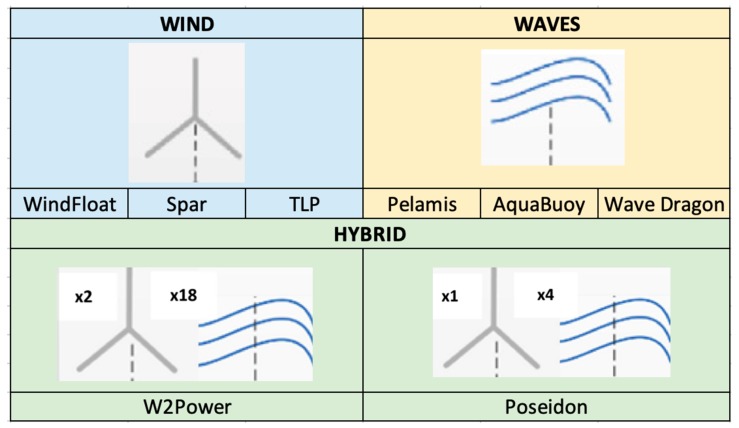
Type of floating platform inputs: wind (WindFlaot, spar, TLP), waves (Pelamis, AquaBuoy, Wave Dragon) and Hybrid (W2Power, Poseidon).

Regarding the type of floating platform, the criteria is that they are divided in wind (WindFloat [31], spar [21] and TLP (Tensioned Leg Platform) [21]), waves (Pelamis [25], AquaBuoy [32] and Wave Dragon [33]) or hybrid (W2Power [28] and Poseidon [34]) (see Figure 4). Depending on the type of platform selected, the protocol to calculate the cost of manufacturing the platform, its generator, its mooring and its anchoring, will change. In addition, it also affects to the installation, maintenance and decommissioning cost.

**Figure 5:**
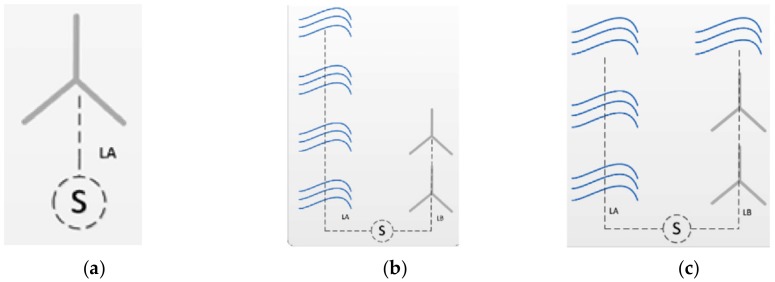

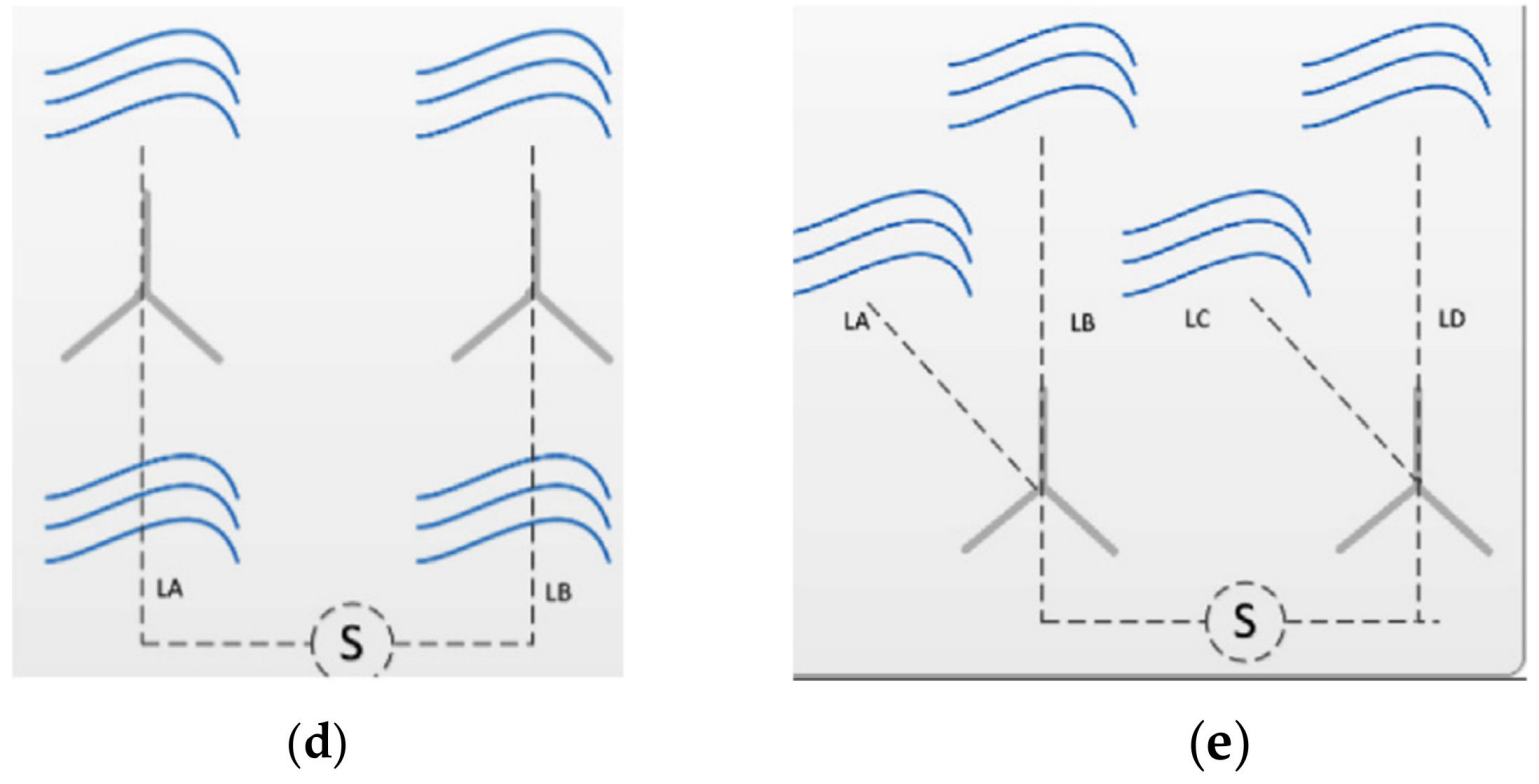
Configuration of the farm inputs [35]: one renewable energy (**a**), two renewable energies and independent arrays (**b**), two renewable energies and peripherally distributed arrays (**c**), two renewable energies and uniformly distributed arrays (**d**), and two renewable energies and non-uniformly distributed arrays (**e**).

Regarding the configuration of the farm figure shows the four types of inputs: one renewable energy (Figure 5a), two renewable energies and independent arrays (Figure 5b), two renewable energies and peripherally distributed array. Figure 5c two renewable energies and uniformly distributed array (Figure 5d) and two renewable energies and non-uniformly distributed array (Figure 5e). They will condition the length, type and size of the electric cable between platforms, which has a great influence on costs.

Considering the type of calculation of the wave’s energy, there are two main types, namely: T and H (period and height of waves, respectively), and matrix. The first consideration takes into account a general equation for the calculation of the energy generated by a wave energy converter considering the period of waves ($T_{wa}$), the height of waves ($H_{wa}$), the number of hours per year (NHAT), the density of water (ρ), the gravity (g), the main dimension ($D_{wa}$), and the efficiency of the wave generator ($\eta_{effiency}$). This equation is used when there is not enough information of the area selected to create the matrix (see Equation (1) [35]). Therefore, this calculation is an approximation to the real value of the energy produced by the wave farm.

(1) $$E_{1wa}=NHAT\cdot \frac{2}{64}\cdot \frac{\rho \cdot g^{2}}{\pi }\cdot T_{wa}\cdot H_{\;wa}^{2}\cdot D_{wa}\cdot \eta_{effiency}$$

The second type considers the power matrix of the technology. Table 3 shows an example of the power matrix of the Pelamis wave energy converter) and the matrix of the location, which is more difficult to obtain. 

On the other side, the power farm and the life-cycle of the farm are introduced by the user, depending on his needs. The electric tariff, capital cost, corporate tax, and steel cost are values that depend on the market being analyzed. The percentage of financing and the interest are determined by the financing company. Finally, the capacity of the shipyard depends on the shipyard where the platforms were built, mainly its size, and works in development in the moment of building the platforms.

Finally, considering the outputs, the equations used are shown in Table 4, as follows [36,37,38]: C1, in €, is the defining cost; C2 in € is the designing and developing cost; C3, in €, is the manufacturing cost; C4, in €, is the installing cost; C5, in €, is the exploiting cost; C6, in €, is the dismantling cost, $C_{t}$, in €, is the cost of the correspondent year, $E_{t}$, in MWh/year, is the energy produced, r, in %, is the capital cost; $CF_{t}$ is the cash flow; t, in years, is the life-cycle of the project; and $G_{0}$, in €, is the initial investment. The project will be economically feasible if the net present value is positive, the internal rate of return is higher than the capital cost, and the levelized cost of energy has low values.

## 3. Case of Study and Results

### 3.1. Case of Study

**Figure 6:**
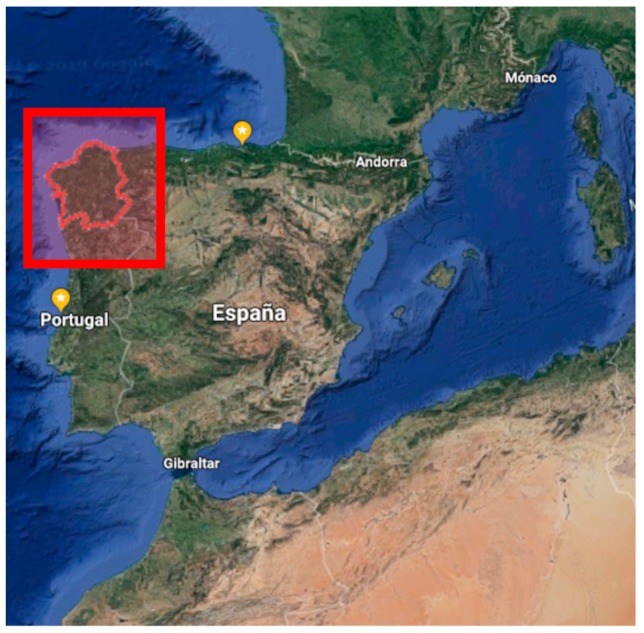
Galicia region (NW of Spain), in red.

The case of study of the present paper is the Galician region, located in the North-West of Spain (in red in Figure 6), which has very good conditions in terms of offshore wind and offshore wave resources.

The four cases studied in this paper differ, depending on their type of floating offshore renewable energy platforms (Table 5), as follows: wind platform (WindFloat) [16,39] in Case 1, wind platform (WindFloat) and wave platform (AquaBuoy) in Case 2, wave platform (AquaBuoy) [26] in Case 3, and a hybrid platform that mixes wind and wave energy in the same platform (W2Power [28]) in Case 4.

For the case study of this paper, the inputs of the four cases of study are shown in Table 6.

### 3.2. Results

**Figure 7:**
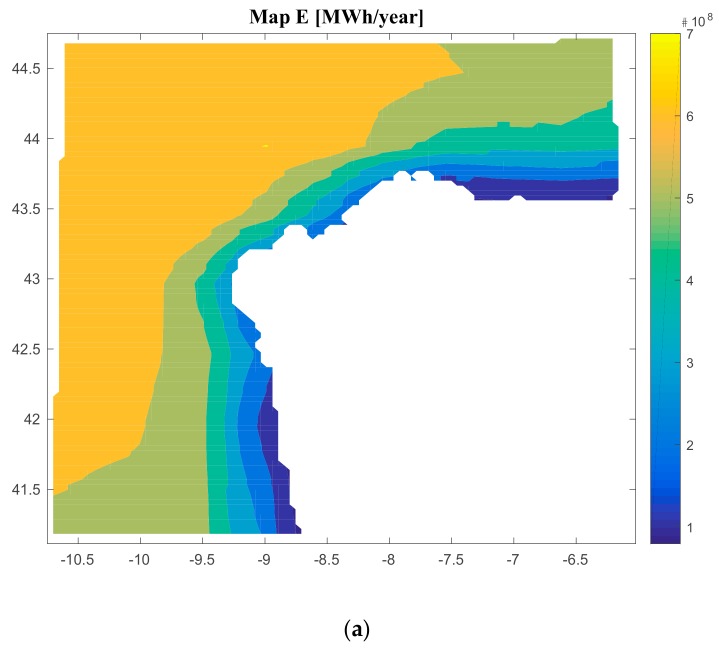

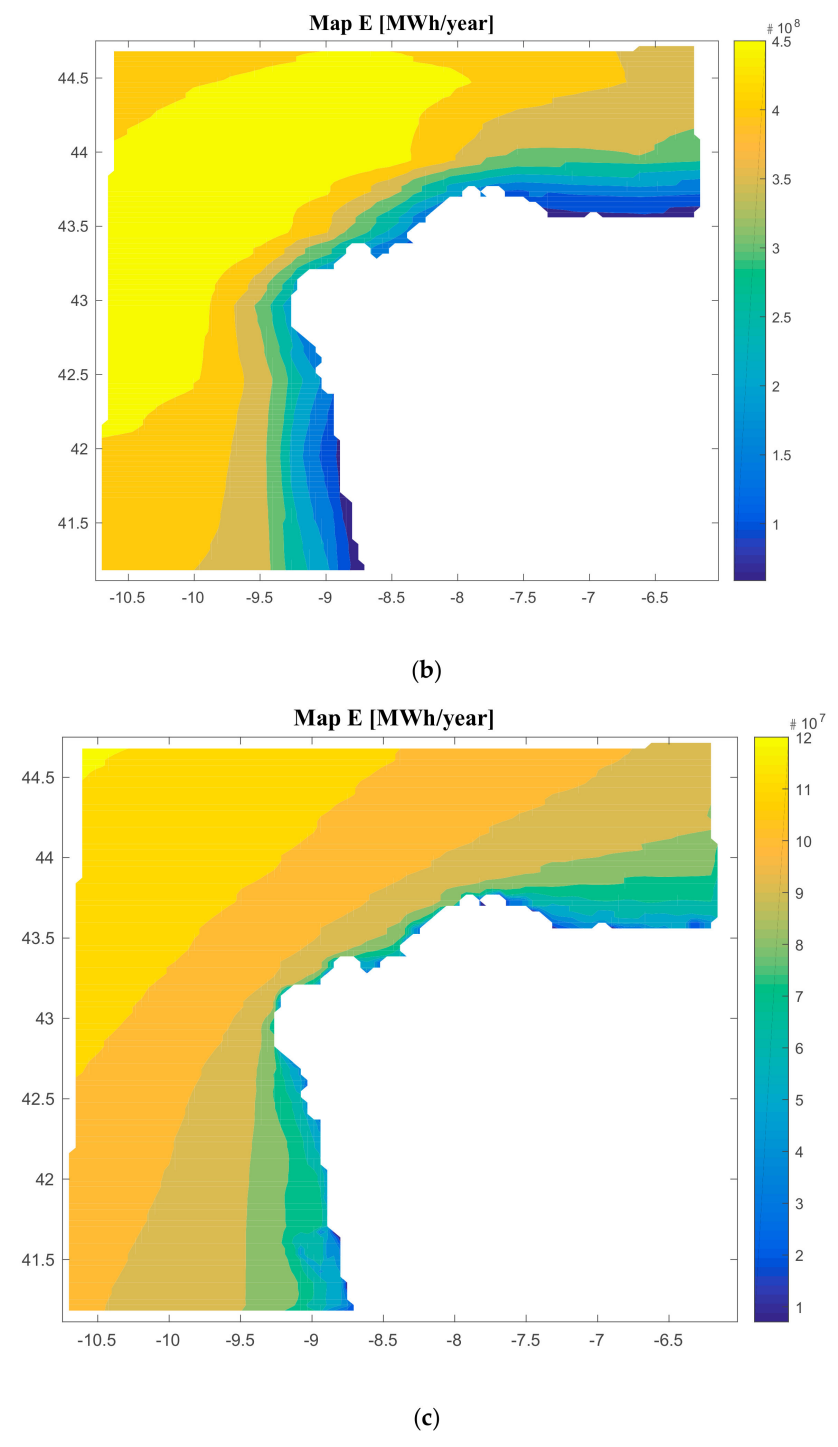

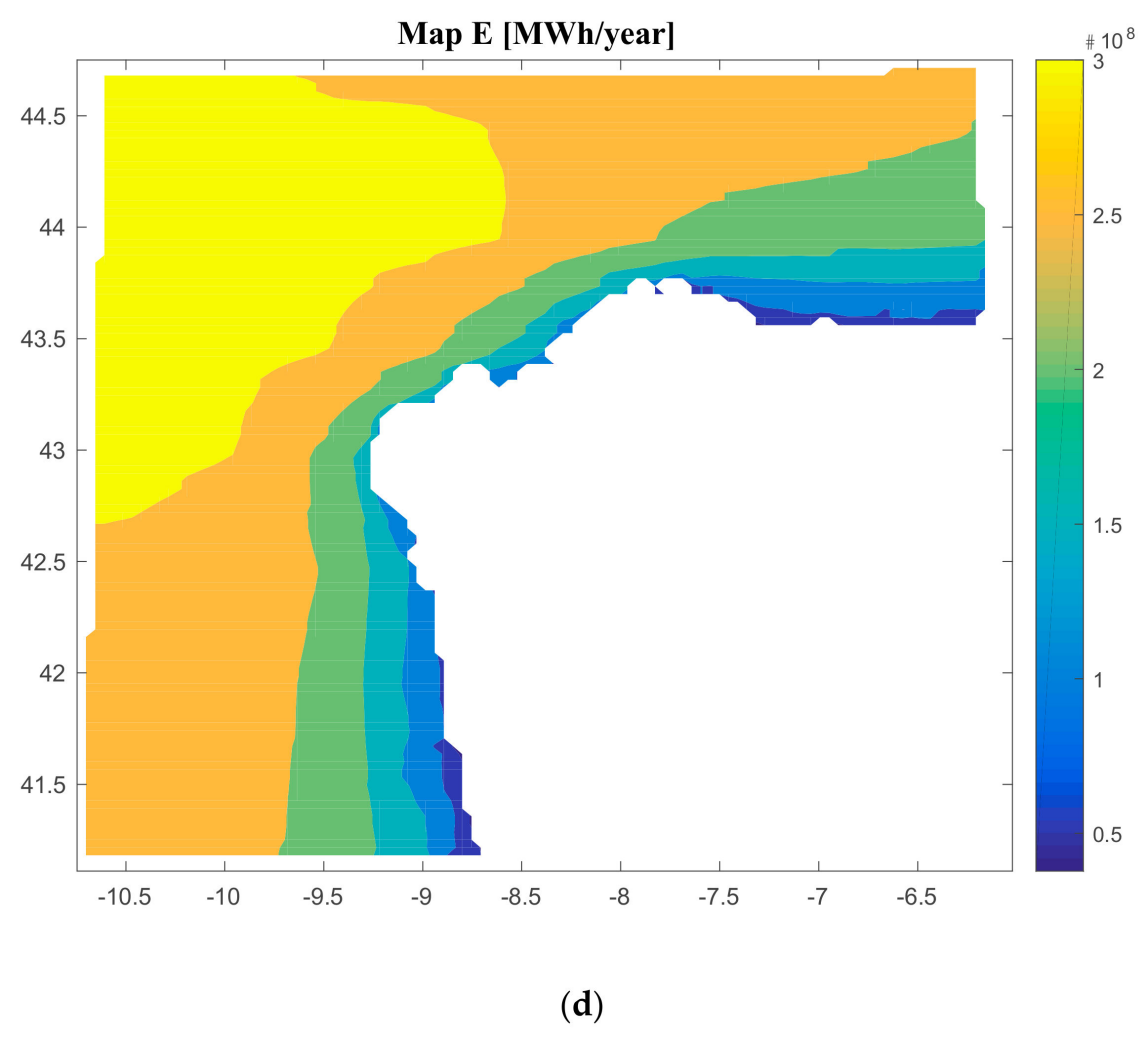
Results of the energy produced for Case 1 (**a**); Results of the energy produced for Case 2 (**b**); Results of the energy produced for Case 3 (**c**); Results of the energy produced for Case 4 (**d**).

The software created calculates the energy produced by a floating offshore renewable energy farm. In the particular cases of study of this paper, the energy produced by a floating offshore renewable energy farm composed by offshore wind turbines (Figure 7a), offshore wind and waves (different platforms) (Figure 7b), offshore wave energy (Figure 7c) and offshore hybrid platform (wind and waves in the same platform) (Figure 7d), has different values depending on the type of technology selected. The values go from 79,500,000 MWh/year to 701,000,000 M€/year for case 1; from 58,890,000 MWh/year to 497,340,000 M€/year for case 2; from 7,087,500 MWh/year to 121,110,000 M€/year for case 3; and from 37,843,000 MWh/year to 312,220,000 M€/year for case 4.

On the other hand, the software gives results regarding the costs (C1, C2, C3, C4, C5, C6, and Ctotal) and the economic feasibility of the floating offshore renewable energy farm (LCOE, IRR, NPV, and DPBP). 

**Figure 8:**
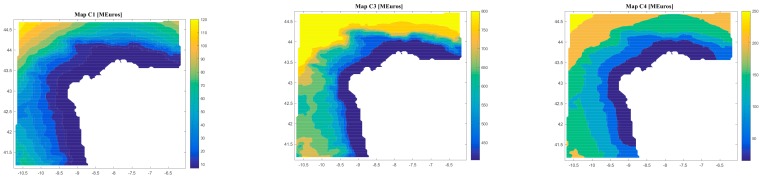

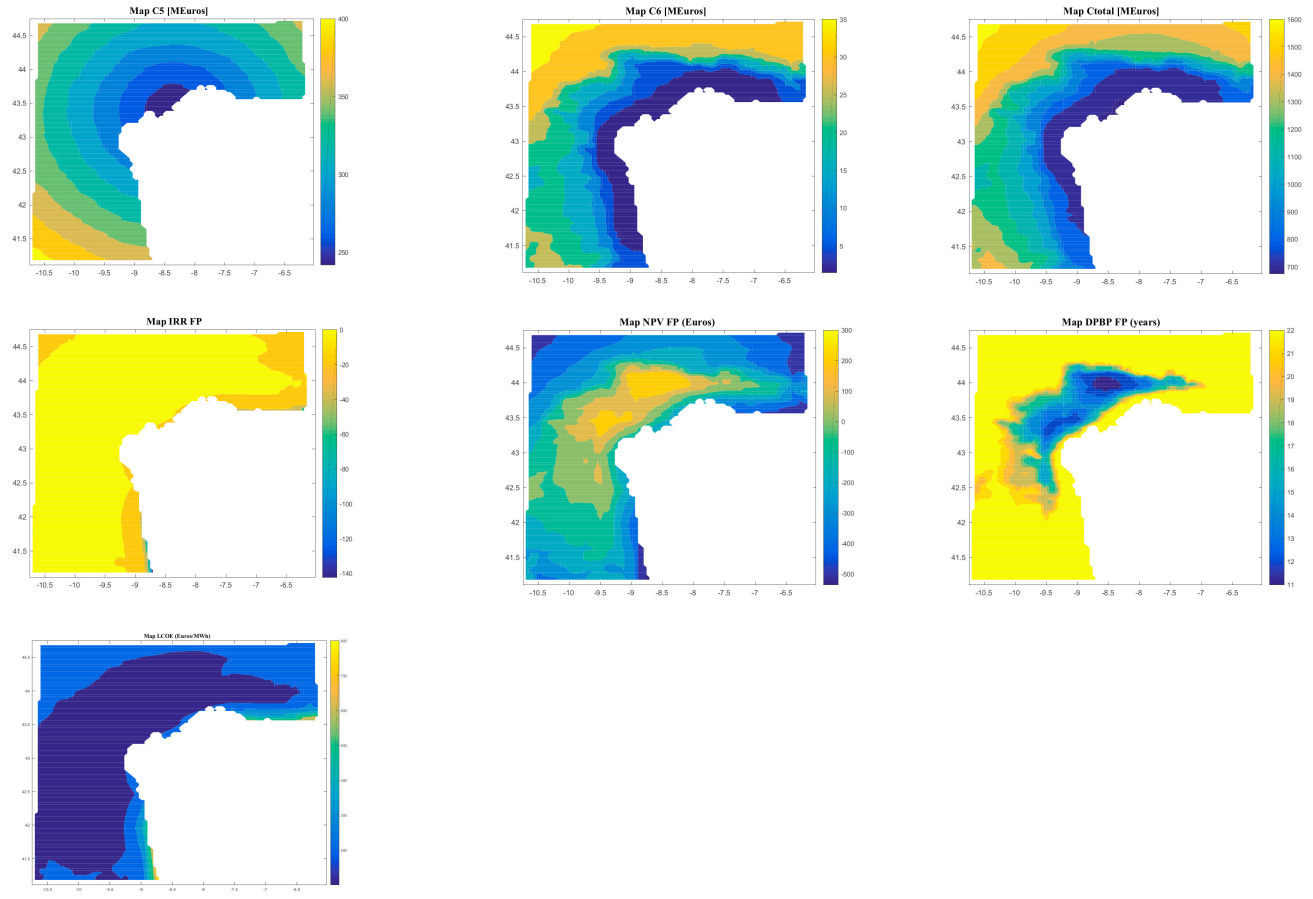
Results (C1, C1, C3, C4, C5, C6, Ctotal, LCOE, DPBP, IRR, NPV) for Case 1.

**Figure 9:**
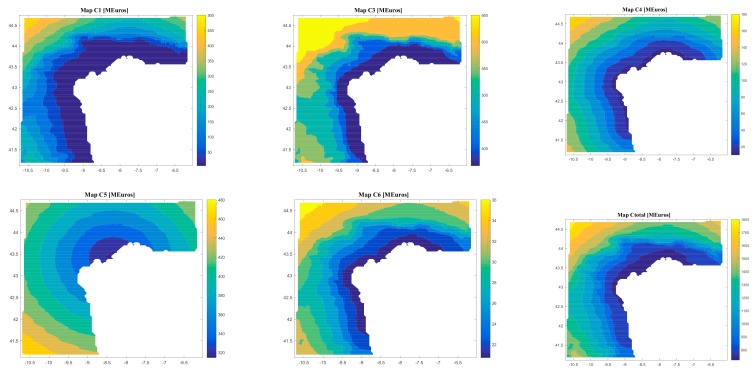

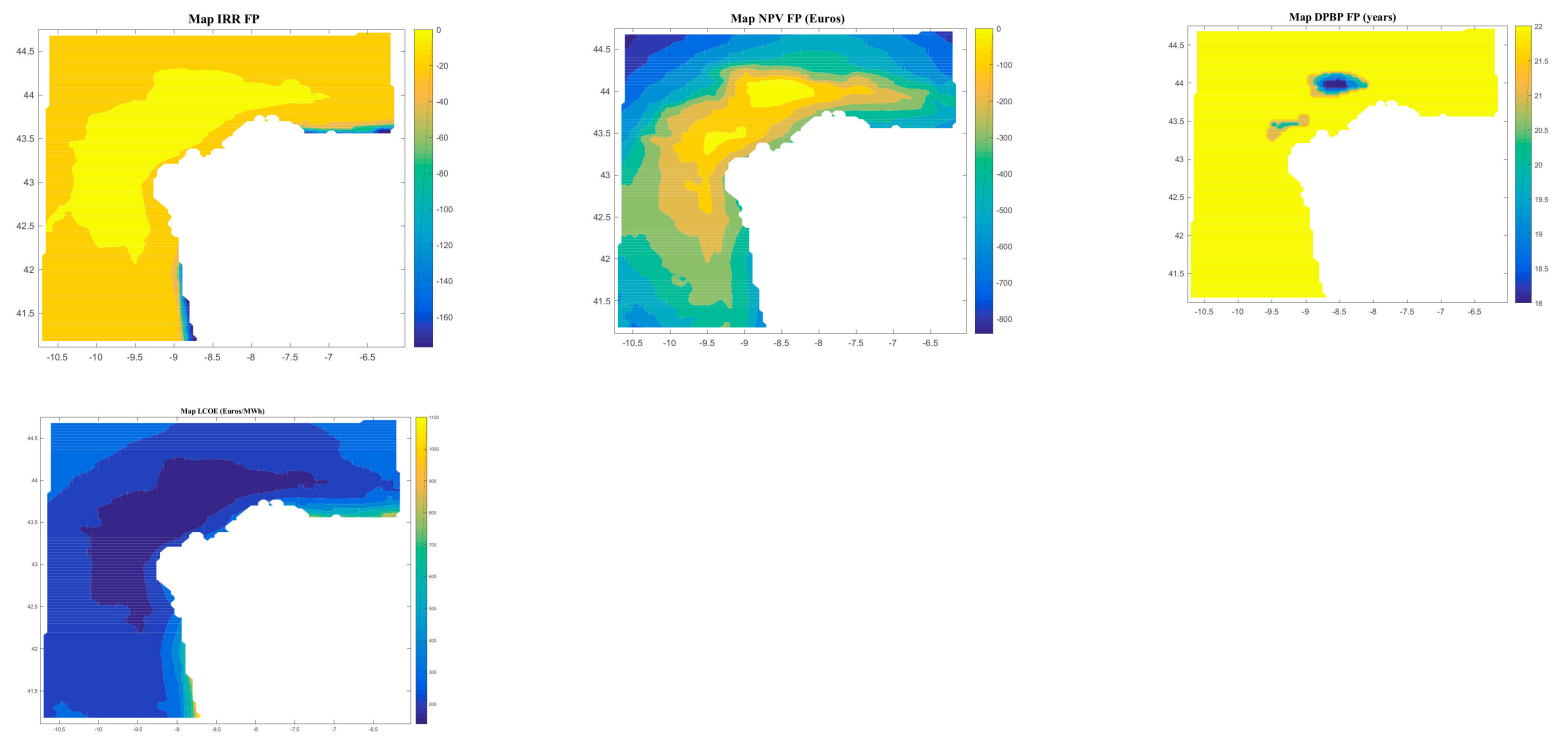
Results (C1, C1, C3, C4, C5, C6, Ctotal, LCOE, DPBP, IRR, NPV) for Case 2.

**Figure 10:**
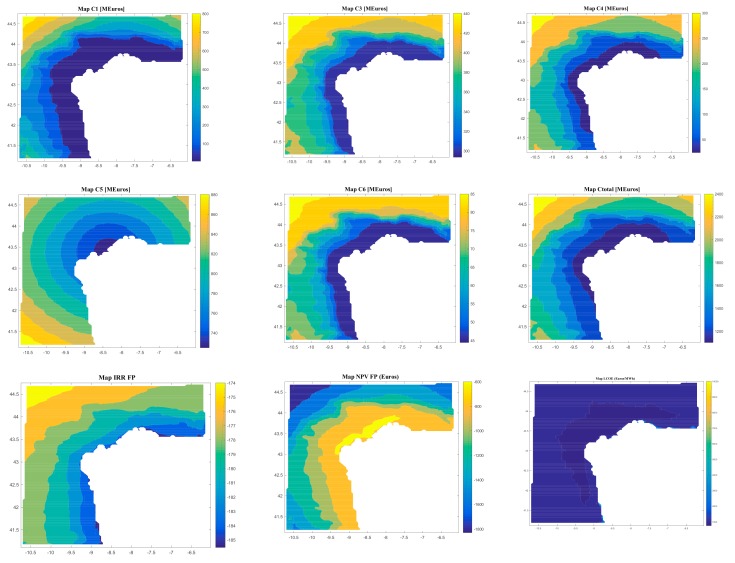
Results (C1, C1, C3, C4, C5, C6, Ctotal, LCOE, DPBP, IRR, NPV) for Case 3.

**Figure 11:**
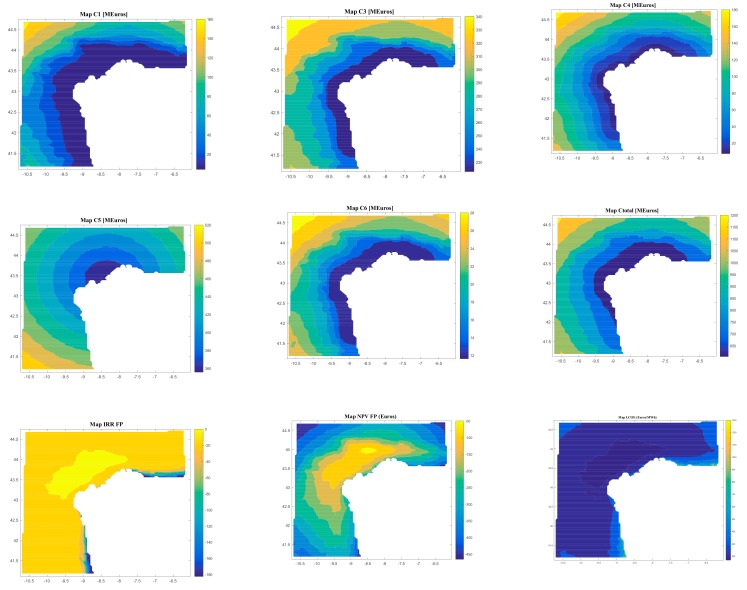
Results (C1, C1, C3, C4, C5, C6, Ctotal, LCOE, DPBP, IRR, NPV) for Case 4.

Figure 8, Figure 9, Figure 10 and Figure 11 are the maps of results for case 1, case 2, case 3 and case 4 respectively. It is important to notice that results of all the maps depend on the shape and scale parameters of the offshore wind resource, the distance from farm to shore, distance from farm to shipyard, distance from farm to port, the height and the period of waves and the bathymetry of the location of study (in this case the Galician region). This fact is shown in Figure 8, Figure 9, Figure 10 and Figure 11. Of course, the maps of results depend on the inputs, therefore this software is valid for all the locations that user wants.

Regarding case 1, results depends on the location and they go (see Figure 8): from 6.94 M€ to 126.52 M€ for C1; 0.46 M€ for C2; from 403.02 M€ to 847.23 M€ for C3; from 14.85 M€ to 272.11 M€ for C4; from 241.68 M€ to 409.15 M€ for C5; from 1.44 M€ to 37.23 M€ for C6; from 674.21 M€ to 1,650 M€ for Ctotal; from 100.31 €/MWh to 882.93 €/MWh for LCOE; from 11 years to 22 years for DPBP; from -142.40% to 14.75% for IRR; and from -535.50 € to 330.85 € for NPV.

Regarding Case 2, the results depend on the location, and they go (see Figure 9) from 4.85 M€ to 503.08 M€ for C1, 0.34 M€ for C2, from 367.45 M€ to 676.09 M€ for C3, from 9.92 M€ to 180.32 M€ for C4, from 314.59 M€ to 482.57 M€ for C5, from 20.72 M€ to 37.46 M€ for C6, from 720.97 M€ to 1835.70 M€ for Ctotal, from 138.22 €/MWh to 1186.30 €/MWh for LCOE, from 18 years to 22 years for DPBP, from −176.90% to 7.83% for IRR, and from −840.31 € to 59.06 € for NPV.

Regarding case 2, results depends on the location and they go (see Figure 9): from 4.85 M€ to 503.08 M€ for C1; 0.34 M€ for C2; from 367.45 M€ to 676.09 M€ for C3; from 9.92 M€ to 180.32 M€ for C4; from 314.59 M€ to 482.57 M€ for C5; from 20.72 M€ to 37.46 M€ for C6; from 720.97 M€ to 1,835.70 M€ for Ctotal; from 138.22 €/MWh to 1,186.30 €/MWh for LCOE; from 18 years to 22 years for DPBP; from -176.90% to 7.83% for IRR; and from -840.31 € to 59.06 € for NPV.

Regarding Case 4, the results depend on the location, and they go (see Figure 11) from 4.14 M€ to 167.97 M€ for C1, 0.17 M€ for C2, from 222.77 M€ to 350.75 M€ for C3, from 7.37 M€ to 186.64 M€ for C4, from 355.56 M€ to 521.30 M€ for C5, from 11.60 M€ to 29.81 M€ for C6, from 603.83 M€ to 1210.40 M€ for Ctotal, from 167.85 €/MWh to 1340.10 €/MWh for LCOE, more than 22 years for DPBP, from −182.39% to −3.57% for IRR, and from −465.38 € to −39.78 € for NPV.

Therefore, considering all of these results, the user can select what is the best technology depending on the location. In this sense, in terms of LCOE, the best technology for the case study is the Case 1 (offshore wind—WindFloat), because it has the smallest LCOE (100.31 €/MWh), and the worst technology is Case 3 (wave energy-AquaBuoy), with a minimum value of 756.39 €/MWh. On the other hand, in terms of IRR and NPV, the best value is also for Case 1 with 14.75% and 330.85 €, respectively, and the worst is Case 3, with values of −173.52% and −581.42 €, respectively.

## 4. Conclusions

The objective of this work was to present a software to calculate the most important parameters of the feasibility of a floating offshore renewable farm in a selected location. This tool can be valuable for enterprises or public entities that want to know the best places where an offshore renewable energy farm can be located, because this can compile the software for the economic calculation of the most important types of offshore renewable energies existing in the world.

The software can calculate the economic parameters of several types of offshore renewable energies, namely: one renewable energy (floating offshore wind—WindFloat, TLP, and spar; floating wave energy—Pelamis and AquaBuoy), hybrid offshore wind and wave systems (Wave Dragon and W2Power), and combined offshore wind and waves with different systems (independent arrays, peripherally distributed arrays, uniformly distributed arrays, and non-uniformly distributed arrays). 

The user can select several inputs, namely: location, configuration of the farm, type of floating offshore platform, type of calculation of the wave’s energy, power of the farm, life-cycle of the farm, electric tariff, capital cost, corporate tax, steel cost, percentage of financing, interest, and capacity of the shipyard. 

The economic results are as follows: the cost of each phase of the life-cycle of the project, the total cost of the life-cycle of the farm, the internal rate of return (IRR), the net present value (NPV), the discounted payback period (DPBP), and the levelized cost of energy (LCOE).

The case study considered here analysed the Galician region (North-West of Spain), using four alternatives depending on the technology, namely: one renewable energy (offshore wind, WindFloat; wave energy, AquaBuoy; and a hybrid system with offshore wind and waves, W2Power) and two renewable energies installed separately in the same farm (offshore wind, Windfloat and wave energy, AquaBuoy). The software gives the results for the case that the user selects. In this particular case study, the technology that is more economically feasible is the offshore wind (WindFloat) with the best value of LCOE (100.31 €/MWh), IRR (14.75%, and NPV (330.85 €), and the technology with the worst economic values is the wave energy (AquaBuoy), with results of 756.39 €/MWh (LCOE), −173.52% of IRR, and −581.42 € of NPV.

The results depend on the shape and scale parameters of the offshore wind resources, the distance from farm to shore, distance from farm to shipyard, distance from farm to port, the height and the period of waves, and the bathymetry of the location of study. The results indicate the economic feasibility of a farm of floating offshore renewable energy in a particular location.

## Figures and Tables

**Table 1:** Inputs of the software I. TLP- tensioned leg platform.

Input	Types
Location	Galicia
	Galicia and Cantabric region
	Portugal
Configuration of the farm	One renewable energy
	Two renewable energies-independent arrays
	Two renewable energies-peripherally distributed array
	Two renewable energies-uniformly distributed array
	Two renewable energies-non-uniformly distributed array
Type of floating platform	WindFloat
	Pelamis
	AquaBuoy
	Wave Dragon
	W2Power
	Poseidon
	TLP
	Spar
Type of calculation of the wave’s energy	T and H
	Matrix

**Table 2:** Inputs of the software II.

Inputs
Power of the farm (MW)
Life-cycle of the farm (years)
Electric tariff (€/MWh)
Capital cost (%)
Corporate tax (%)
Steel cost (€/ton)
Percentage of Financing (%)
Interest (%)
Capacity of the shipyard (platform/year)

**Table 3:** Pelamis power matrix (in kW) [36].

Te(s)Hs(m)	Power Matrix (in kW)																
	5	5.5	6	6.5	7	7.5	8	8.5	9	9.5	10	10.5	11	11.5	12	12.5	13
0.5	0	0	0	0	0	0	0	0	0	0	0	0	0	0	0	0	0
1	0	22	29	34	37	38	38	37	35	32	29	26	23	21	0	0	0
1.5	32	50	65	76	83	86	86	83	78	72	65	59	53	47	42	37	33
2	57	88	115	136	148	153	152	147	138	127	116	104	93	83	74	66	59
2.5	89	138	180	212	231	238	238	230	216	199	181	163	146	130	116	103	92
3	129	198	260	305	332	240	332	315	292	266	240	219	210	188	167	149	132
3.5	0	270	345	415	438	440	424	404	377	362	326	292	260	230	215	202	180
4	0	0	462	502	540	546	530	499	475	429	384	366	339	301	267	237	213
4.5	0	0	544	635	642	648	628	590	562	528	473	432	382	356	338	300	266
5	0	0	0	739	726	726	707	687	670	607	557	521	472	417	369	348	328
5.5	0	0	0	750	750	750	750	750	737	667	658	586	530	496	446	395	355
6	0	0	0	0	750	750	750	750	750	750	711	633	619	558	512	470	415
6.5	0	0	0	0	750	750	750	750	750	750	750	743	658	621	579	512	481
7	0	0	0	0	0	750	750	750	750	750	750	750	750	676	613	584	525
7.5	0	0	0	0	0	0	750	750	750	750	750	750	750	750	686	622	593
8	0	0	0	0	0	0	0	750	750	750	750	750	750	750	750	690	625

**Table 4:** Output criteria.

Output	Equation
Total cost	$C_{total}=C1+C2+C3+C4+C5+C6$
Levelized cost of energy	$LCOE=\frac{\sum_{t=0}^{N_{farm}}\frac{C_{t}}{{\left(1+r\right)}^{t}}}{\sum_{t=0}^{N_{farm}}\frac{E_{t}}{{\left(1+r\right)}^{t}}}$
Net present value	$NPV=-G_{0}+\overset{n}{\underset{t=1}{\sum }}\frac{CF_{t}}{{\left(1+r\right)}^{t}}$
Internal rate of return	$-G_{0}+\sum_{t=1}^{n}\frac{CF_{t}}{{\left(1+IRR\right)}^{t}}=0$

**Table 5:** Characteristics of the case studies.

Input	Case of Study 1	Case of Study 2	Case of Study 3	Case of Study 4
Location	Galicia	Galicia	Galicia	Galicia
Configuration of the farm	One Renewable Energy	Two Renewable Energies–Independent Arrays (IA)	One Renewable Energy	One Renewable Energy
Floating platform	WindFloat	WindFloat and AquaBuoy	AquaBuoy	W2Power
Calculation energy waves	-	T and H	T and H	T and H

**Table 6:** Inputs.

Input	Value	Units
Power of the farm	200	MW
Life-cycle of the farm	20	years
Electric tariff	150	€/MWh
Capital cost	8%	-
Corporate tax	25%	-
Steel cost	524	€/ton
Percentage of financing	60%	-
Interest	5.24%	-
Capacity of the shipyard	5	Platforms/year
